# Visualization of chemical bonding in a silica-filled rubber nanocomposite using STEM-EELS

**DOI:** 10.1038/s41598-020-78393-0

**Published:** 2020-12-09

**Authors:** Yohei K. Sato, Yasufumi Kuwauchi, Wakana Miyoshi, Hiroshi Jinnai

**Affiliations:** 1 Institute of Multidisciplinary Research for Advanced Materials, Tohoku University, 2-1-1 Katahira, Aoba-ku, Sendai, Miyagi 980-8577 Japan; 2 Sumitomo Rubber Industries, Ltd, 1-1, 2-chome, Tsutsui-cho, Chuo-ku, Kobe, 651-0071 Japan; 3 Present Address: Toray Research Center, Inc., 3-3-7, Sonoyama, Otsu, Shiga 520-8567 Japan

**Keywords:** Materials science, Nanoscience and technology

## Abstract

In nanocomposites, the adhesion between nanofillers and the polymeric matrix is key to the mechanical properties. The strength and spatial distribution of the adhesive layer around the nanofillers are important, particularly the presence of chemical bonding between the nanofillers and matrix. In this work, we studied a styrene-butadiene rubber composite filled with silica nanoparticles to visualize the spatial distribution of the adhesive layer. A silane coupling agent (SCA) was added to the nanocomposite for strong adhesion. The reaction involving the SCA on the silica surface was investigated by scanning transmission electron microscopy combined with electron energy-loss spectroscopy. Si-L_2,3_ spectra of the silica-filled rubber nanocomposite without the SCA were the same around the nanofillers, whereas in the nanocomposite containing the SCA the spectra were position-dependent. The spectra were fitted with the intensity profiles of the Si-L_2,3_ spectra of silica and SCA by multiple linear least-squares fitting. The fitting coefficients of silica and SCA were used to map the spatial distribution of the chemical bonding between silica and rubber chains. Chemical bonding was observed around the silica nanoparticles but not in the SBR matrix region, providing direct evidence of the reinforcing mechanism in the silica-filled rubber nanocomposite.

## Introduction

Organic–inorganic nanocomposites are a class of polymer-based multicomponent materials that have been attracting considerable interest because they often exhibit unexpected properties that arise synergistically from the constituents^1–3^. Nanofillers form hybrids with organic polymers, and increase the static and dynamic moduli, strength, and thermal and electrical conductivities of the polymers^4,5^. A common example of the superior mechanical properties of nanocomposites is tread rubber, which requires (wet) grip performance and low-rolling resistance for low fuel consumption.

Silica nanoparticles are a typical filler used with cross-linked rubber. These rubber nanocomposites have non-linear mechanical properties, including a strain-dependent dynamic modulus^6,7^, high hysteresis^8^, and stress softening^9,10^. Because the silica surface is highly polar and hydrophilic, it is less compatible with non-polar rubber. A silane coupling agent (SCA) is often used to improve the compatibility between the silica and rubber. The SCA forms covalent bonds between the silica filler and rubber, which affects the mechanical properties of the rubber nanocomposite substantially. Therefore, the effect of the SCA covalent bonds at the silica/rubber interface on the mechanical properties of the tire rubbers has been studied extensively.

The effects of SCAs in silica-filled rubber nanocomposites have been examined by measuring the dynamic modulus^11–15^, which depends strongly on the amount of the SCA. In contrast, Qu et al.^16^ reported that the amount of the SCA did not affect the dynamic modulus. They suggested that the SCA bound not only to the silica surface, but also to the rubber chains. Therefore, it is essential to evaluate whether the SCAs react efficiently on the silica surfaces when the rubber is mixed with the other ingredients. The interfacial reaction between silica and rubber can be followed by spectroscopic techniques such as X-ray photoelectron spectroscopy^17,18^, X-ray-induced Auger electron spectroscopy^17^, NMR^19,20^, and infrared spectroscopy^21,22^. These studies showed that O–Si–C covalent bonds with the SCA were present in the rubber. Although these spectroscopic methods are efficient, they give spatially averaged information and do not reveal the local bonding states at the silica interface.

Transmission electron microscopy (TEM) can provide information about local detailed structures, such as the interface between silica fillers and rubber. The filler-to-filler and filler-to-rubber interactions have been observed by TEM^9^. Voids around silica nanoparticles were observed in a silica-filled rubber nanocomposite without an SCA, whereas that with an SCA had no voids, suggesting strong filler-to-rubber interactions. Similarly, analytical TEM combined with electron energy-loss spectroscopy (EELS) visualizes the element map of a nanocomposite^23^. Energy-filtered TEM was used to study the distribution of S-containing SCA in a silica-filled rubber nanocomposite by detecting the intensities of the Si-L_2,3_ edge (135 ± 10 eV) and S-L_2,3_ edge (175 ± 10 eV). It was assumed that the intensities of the Si-L_2,3_ edge contained only the Si distribution, whereas those of the S-L_2,3_ edge contained both the S and Si distribution. The spatial distribution of S was obtained from the intensity ratio of the S-L_2,3_ to Si-L_2,3_ edge, which was found around the silica nanoparticles. However, in practice, rubber composites are often vulcanized to increase the strength of the rubber composite and the S atoms are distributed throughout the rubber matrix rather than just around the SCA. Therefore, the S distribution may not be an appropriate marker for the SCA in rubber nanocomposites.

Scanning transmission electron microscopy (STEM)-EELS is a powerful method for probing the local bonding states in materials^22,24^. The spectral intensity distribution of the electron energy-loss near-edge structure (ELNES), which corresponds roughly to the density of states in the conduction band (unoccupied states), reflects the chemical bonding states. In addition, ELNES can be used as a fingerprint for mapping the chemical bonding of the material^22,24,25^. Therefore, it is important to identify the ELNES spectra that are characteristic of the interface interaction in silica-filled rubber to visualize directly the chemical bonding states between silica and rubber due to SCA.

In this study, Si-L_2,3_ spectra of silica nanoparticles in a styrene-butadiene rubber (SBR) matrix were measured using STEM-EELS to investigate the chemical bonding states at the interface between silica and SBR with and without SCAs. The spectral profiles of silica and SCA were used as fingerprints for the chemical bonding states. The spatial distributions of the SCA bonds in SBR were visualized for silica-filled SBR nanocomposites containing various amounts of SCA.

## Results and discussions

**Figure 1:**
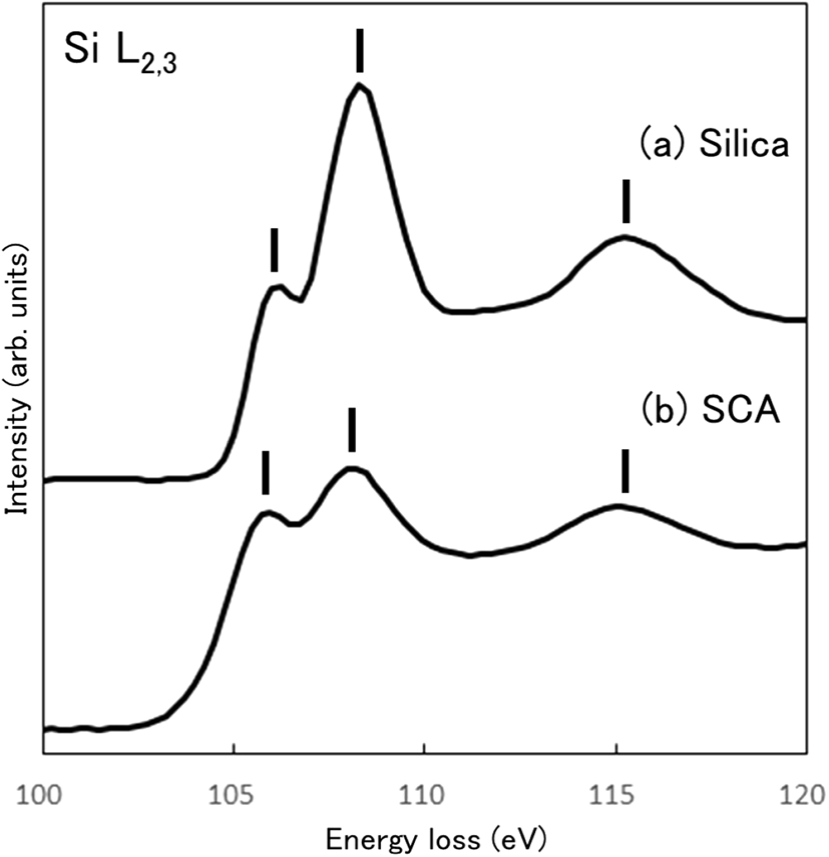
Si-L_2,3_ edge EELS spectra of **(a)** silica and **(b)** SCA.

Figure 1a,b show the spectral intensity profiles of the Si-L_2,3_ edges of the silica and SCA, respectively. The profiles correspond to the partial density of states with *s*- and *d*-symmetries of unoccupied states. Each spectral intensity was normalized to the integral intensity at 100–120 eV. Three characteristic peaks were observed at 106.2, 108.4, and 115.4 eV in the silica spectrum. The spectrum of SCA also showed three peaks at the similar energy positions at 106.0, 108.3, and 115.2 eV. These peaks were assigned as unoccupied electronic states in SiO_4_^4–^ tetrahedron clusters based on molecular orbital calculations^26,27^. Similar spectral profiles have been reported in the XANES^28,29^ and ELNES spectra of α-quartz^30,31^, amorphous quartz^30–32^, and Si(OCH_3_)_4_ in the gas phase^33^, which consist of tetrahedral SiO_4_^4-^ units. The three peaks in the SCA spectrum were observed at almost the same energies with those of silica, indicating that the silicon atom in SCA is also bound in a tetrahedral cluster. The nearest neighbors of the Si atom in SCA are one carbon and three oxygen atoms. Therefore, the chemical bonding states in SCA are slightly different from those in silica and the peaks at 108.3 and 115.4 eV were weaker and broader than those in the silica spectrum. In addition, the onset energy of the SCA spectrum was 102.5 eV, which was 2 eV lower than that of the silica spectrum.

The silica surface modified by SCA in the rubber matrix also has been investigated by XPS, in which the binding energy of Si-2p core levels of the silica surfaces modified by SCA was higher than that of the normal silica^17^. Because the Si-L_2,3_ spectrum corresponds to the excitation from 2p to the unoccupied states, Si-L_2,3_ spectrum of SCA should be shifted to lower energy than that of the silica. The spectrum of the SCA was indeed located at 0.2 eV lower than the silica, which agrees with the results of XPS.

**Figure 2:**
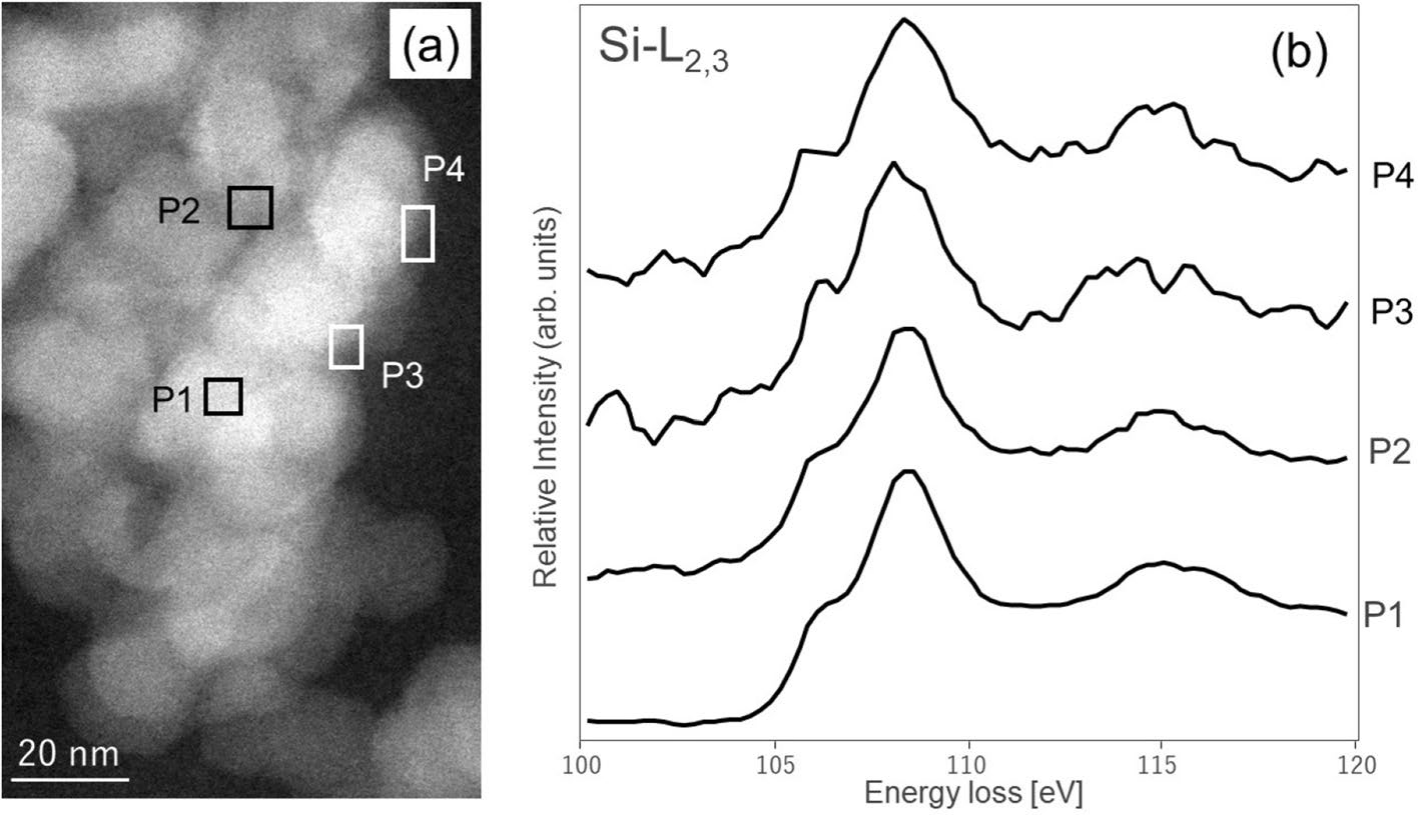
Si-L_2,3_ edge obtained from the SBR composite containing 0 vol % SCA. **(a) **ADF-STEM image and **(b)** Si-L_2,3_ edge EELS spectra. Rectangles P1–P4 in **(a)** indicate the areas from which the spectra in **(b) **were taken. Spectra P1 and P2 are obtained from the silica nanoparticle aggregates. Spectra P3 and P4 are obtained from the particle edges.

Figure 2a shows the annular darkfield (ADF)-STEM image of the silica nanoparticle aggregates in the SBR matrix without the SCA. The contrast of the ADF-STEM image is approximately proportional to Z^~1.7^, where Z is an atomic number^34^. The white and dark contrasts in the ADF-STEM image correspond to the silica nanoparticles and SBR matrix, respectively. Figure 2b shows the Si-L_2,3_ EELS spectra obtained from the rectangular areas labeled P1–P4 in the ADF-STEM image. The spectral intensities were normalized to the intensity integrated over 100–120 eV. All spectra had peaks at 108.2 and 115.0 eV, and shoulders at 106.2 eV. The spectra were similar regardless of the positions from which they were obtained, and they were similar to the profile of silica in Fig. 1. Therefore, the chemical bonding states of the silicon atoms at the surface and inside the nanoparticles were similar in the SBR composites without the SCA.

**Figure 3:**
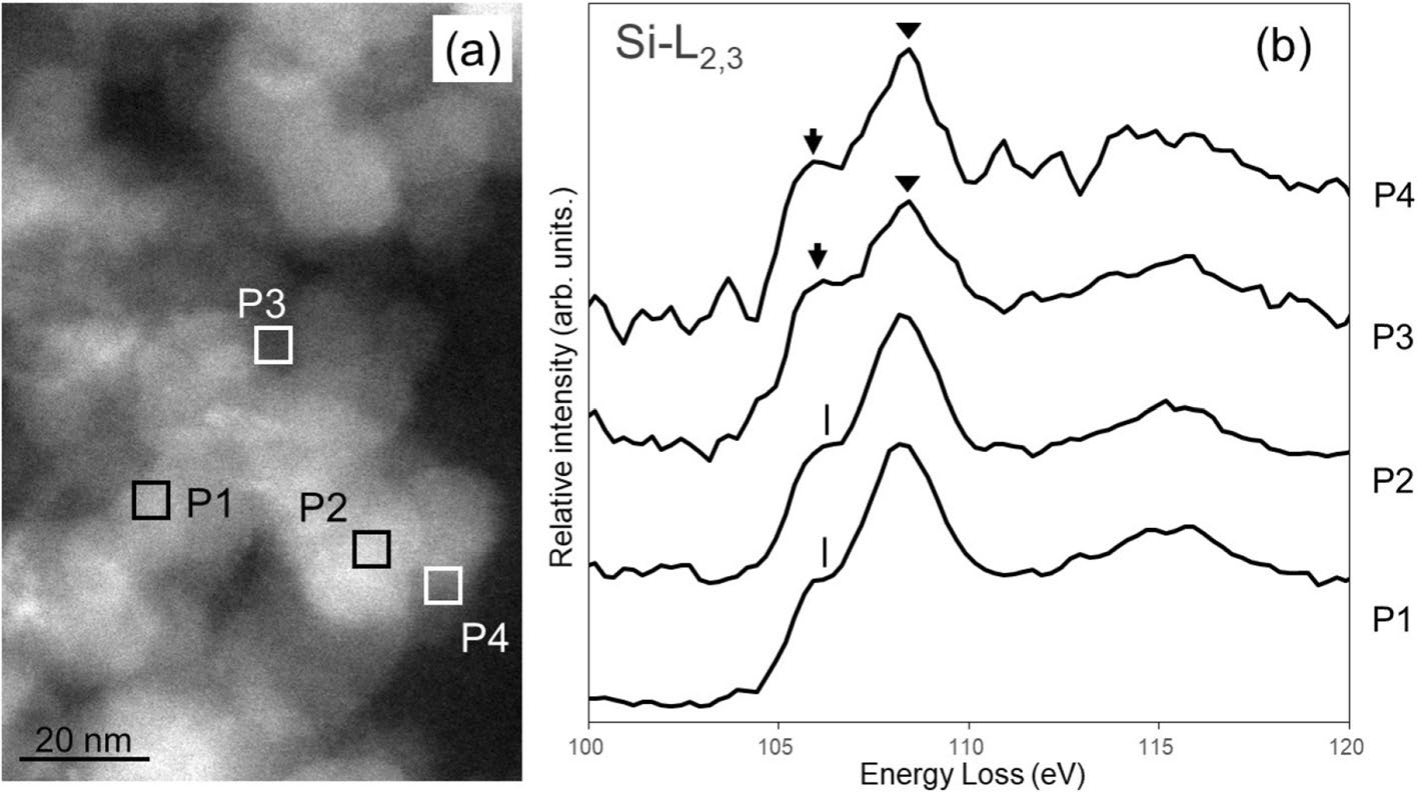
Si-L_2,3_ edge of the SBR composite containing 17 vol % SCA. **(a)** ADF-STEM image and **(b)** Si-L_2,3_ edge EELS spectra. Rectangles P1–P4 in **(a)** indicate the areas from which the spectra in **(b)** were taken. Spectra P1 and P2 are obtained from the silica nanoparticle aggregates. Spectra P3 and P4 are from the particle edges. The spectral distributions at the silica interface are broader than those of the aggregates.

Figure 3a shows the ADF-STEM images of silica nanoparticles in SBR containing 17 vol % SCA. Figure 3b shows the Si-L_2,3_ spectra obtained from rectangular areas P1–P4 in Fig. 3a. The spectral intensities were normalized to the intensity integrated over 100–120 eV. The spectra taken from P3 and P4 had a low signal-to-noise ratio because the measurement volumes at P3 and P4 were small. The Si-L_2,3_ spectra in Fig. 3 were obtained under the same experimental conditions as the SBR nanocomposite without SCA. Nevertheless, the Si-L_2,3_ spectra in Fig. 3 showed differences between the inside (P1 and P2) and surface (P3 and P4) of the silica nanoparticles. The relative intensity of the shoulder at 106.0 eV (arrows) to that at 108.2 eV (triangle) increased. The peaks at P3 and P4 (indicated by the arrows) were located at slightly lower energies than the peaks at 106.2 eV at P1 and P2 (shown by the vertical lines), reflecting the chemical shift of the bonding state at the interface between the silica and the rubber. Besides, the peak at 115 eV of P3 and P4 were broader than those at P1 and P2. This observation indicated that the bonding state of Si at the interface between the silica and the SBR matrix was different in the SBR nanocomposite containing 17 vol % SCA relative to that inside the silica nanoparticles. The spectral profiles obtained from the interface at P3 and P4 were similar to that of SCA shown in Fig. 1b, with similar relative intensity of the dominant peaks at 106 and 108 eV. This difference in the spectral profiles reflected the interfacial reaction due to the presence of SCA.

The Si-L_2,3_ spectra in Fig. 3b consisted of the silica and the SCA spectra. We decomposed these components from the experimental spectra by using multiple linear least-squares (MLLS) fitting^35^. The Si-L_2,3_ spectra in Fig. 3b were regarded as linear combinations of the silica and SCA reference spectra (Fig. 1). The fitting spectral intensity is described as

1 $${S}_{\mathrm{fit}}\left(E\right)={c}_{\mathrm{silica}}{S}_{\mathrm{silica}}\left(E\right)+{c}_{\mathrm{SCA}}{S}_{\mathrm{SCA}}\left(E\right)$$

where *S*_fit_(*E*), *S*_silica_(*E*), and *S*_SCA_(*E*) are the intensity profiles of the fitting spectrum, silica, and SCA, respectively, *E* is the energy loss, and *c*_silica_ and *c*_SCA_ are the weighting coefficients of the silica and SCA reference materials, respectively. The weighted intensities of the individual reference spectra correspond to the amounts of silica and SCA in the specimen. The relative amount of the two components were visualized by plotting the integrated intensity at each pixel of the STEM-EELS image.

**Figure 4:**
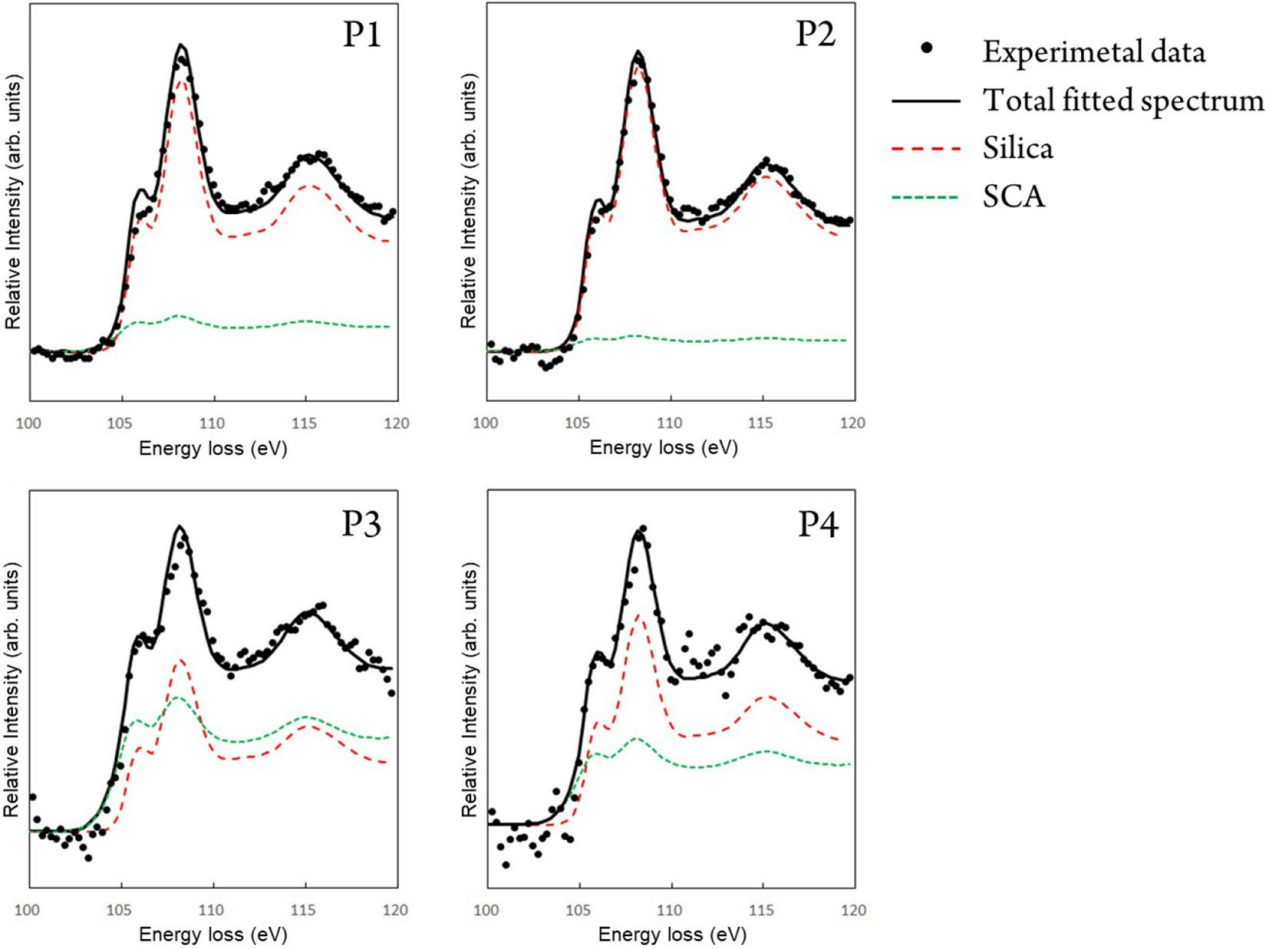
Fitting of the Si-L_2,3_ EELS spectra of the SBR composite containing 17 vol % SCA. The dots and solid lines show the experimental and total fitted spectra, respectively. The red dashed and greed dotted lines are the contributions of the reference spectra of silica and SCA to MLLS fitting, respectively.

Figure 4 shows the fitting results of the spectra obtained from P1–P4 in Fig. 3a. Because the interface regions of the silica nanoparticles (P3 and P4) had small measurement volumes, the intensities of the Si-L_2,3_ spectra from P3 and P4 were noisy due to insufficient statistics. The intensity profiles of the total fitted spectra agreed well with the individual experimental spectra (Fig. 4). The spectra from the agglomerated particles (P1 and P2) showed a high weighted intensity for the silica reference spectrum, whereas the spectra from the particle interfaces (P3 and P4) showed an increase in the SCA component. These results suggest that the interpretation of the Si-L_2,3_ spectra in Fig. 3 as a linear combination of silica and SCA was valid. The experimental peaks around 115 eV in P3 and P4 seems to be slightly broader than those of the fitting spectra, demonstrating that the peak at 115 eV was not simply reproduced by the combination of the reference spectra of the silica and SCA. The previous theoretical studies revealed that the spectral profile around 115 eV for the silica is heavily affected by the second nearest neighbors^26,28^. Therefore, the broadening of spectral profiles around 115 eV for P3 and P4 indicates the formation of characteristic bonding states at the interface between the silica and the rubber.

The weighted intensities of the reference spectra at a pixel correspond to the amount of the individual reference materials. Therefore, we constructed chemical bonding maps. We here note that the present study utilizes electronic state of Si atoms, i.e., the chemical bonding state of Si atoms, instead of the elemental distribution of specific atom, e.g., S in the previous study^23^. The MLLS fittings were conducted for the SBR nanocomposite without SCA. Ideally, no SCA contribution to the fitting should be found, that is, *c*_*SCA*_ should be zero everywhere in the image. However, we found a finite value for *c*_*SCA*_ due to the experimental uncertainty of the spectral measurements. This finite value from the MLLS fittings is an error that should not be included in the chemical imaging. Thus, the maximum value of the spectral intensity of the SCA for the SBR nanocomposite with 0 vol % SCA was defined as the cut-off value, below which *c*_*SCA*_ was considered to be negligibly small in the subsequent chemical bonding map of the nanocomposites containing SCA.

**Figure 5:**
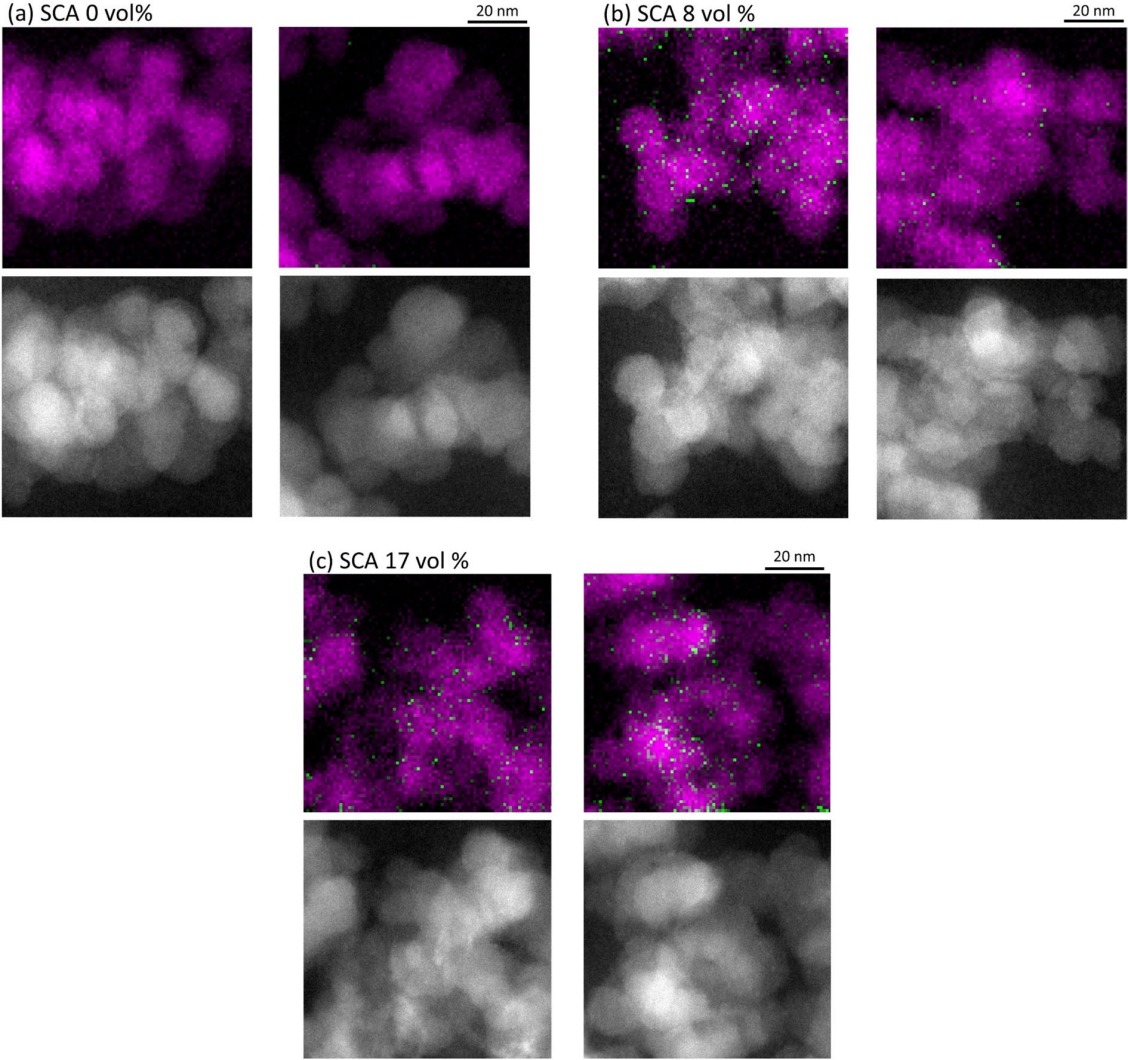
Chemical bonding maps (top) and ADF images (bottom) of silica-filled SBR containing **(a)** 0, **(b)** 8 and **(c)** 17 vol % SCA. Magenta regions and green dots correspond to the bonding states of silica and SCA, respectively.

Figure 5 shows the chemical bonding maps of the SBR nanocomposites containing 0, 8 and 17 vol % SCA from two randomly chosen areas of each nanocomposite. The ADF-STEM images corresponding to the chemical bonding maps are also shown for comparison. The SCA forms covalent bonds between the silica fillers and SBR; thus, the spatial distribution of SCAs is directly correlated with the formation of chemical bonds between the silica nanoparticles and SBR matrix. SCA was observed around the silica nanoparticles but not in the SBR matrix region, indicating that SCA was preferentially adsorbed on the silica nanoparticles.

The 8 vol % SCA SBR nanocomposite showed different densities of SCA around the silica nanoparticles, indicating that the SCA in the nanocomposite was heterogeneously distributed around the nanoparticle’s surface (Fig. 5b). In contrast, in the 17 vol % SCA SBR nanocomposite, the SCA was distributed uniformly around the silica nanoparticles (Fig. 5c). As expected, more SCA was observed in the 17 vol % SCA nanocomposite than in the 8 vol % SCA nanocomposite.

Based on these results, the SCA in the SBR nanocomposites formed chemical bonds between the silica and the SBR, increasing the adhesion at the interface. The 17 vol % SCA nanocomposite showed a uniform distribution of SCA around the silica nanoparticles, indicating increased bonding between the silica nanoparticles and SBR matrix compared with the 8 vol % SCA nanocomposite.

Because the intensity of the Si L_2,3_ spectrum is proportional to the amount of Si atoms, the amount of Si atoms in the SCA with respect to the silica can be estimated from the intensities of the mapping images. The ratios of the intensity of the SCA to that of silica in Fig. 5 were evaluated to be 0.06 and 0.09 for 8 vol % and 17 vol % SCA nanocomposites, respectively, which showed clear correlation with the amount of SCA. We note here that the ratio of Si atoms in the SCA to those in silica is estimated to be 0.01 and 0.02 for the 8 vol % and 17 vol % SCA nanocomposites, respectively. Although these estimated values are substantially lower than the observed ones, this is probably due to insufficient statistics. For more quantitative discussions, EELS measurements in wider area of nanocomposite are required in the future.

## Conclusions

The chemical bonding in SBR nanocomposites containing 8 and 17 vol % SCA were visualized directly by using STEM-EELS. The intensity profiles of the Si-L_2,3_ spectra for the nanocomposites with and without SCAs were obtained. The spatial distribution of SCA, which contributes to the adhesion between the silica nanoparticles and SBR matrix, was visualized directly using STEM-EELS. SCA was preferentially adsorbed on the silica surface and did not bind to the SBR chains. The chemical bonding map of the SBR nanocomposite containing 8 vol % SCA showed heterogeneous SCA distribution around the silica nanoparticles, whereas the SCA uniformly covered the silica nanoparticles in the 17 vol % SCA nanocomposite. The SCA density in the chemical bonding map was correlated with the amount of SCA in the silica-filled SBR nanocomposite. The spatial distribution of the chemical bonding due to SCA at the nanofiller interface should reflect the macroscopic mechanical properties of SBR nanocomposites, and thus STEM-EELS may be useful for developing the high-performance tire rubbers.

## Methods

### Sample preparation

The SBR composites consisted of SBR (Nipol NS 616, Zeon Chemicals) filled with 57.1 vol % silica nanoparticles (ULTRASIL VN 3, Evonik Industries AG) with respect to SBR. The average size of the silica nanoparticles was ~ 20 nm based on TEM observations. Bis(triethoxysilylpropyl)polysulfide (Si266, Evonik Industries AG) was used as an SCA. The amounts of the ingredients are listed in Table 1. Small amounts of other ingredients (oil, wax antioxidant, ZnO, stearic acid, sulfur, and accelerators) were also included in the composites. Three SBR composites containing 0, 8, and 17 vol % SCA with respect to the silica loading were prepared by the same procedure. The particles and the ingredients were added to raw SBR, and then the composites were mixed in an internal mixer. The composites were vulcanized at 150 °C for 12 min. The specimens for STEM-EELS measurements were prepared using a cryo-ultramicrotome. The average thicknesses of the specimens were 80 nm, which were evaluated by electron tomography^36^.

**Table 1:** SBR composite formulation.

Components	Volume percent
SBR	100
Silica	32.0
SCA	0, 8.5, 17.1 vol % with respect to filler

The composites were vulcanized at 150 °C for 12 min. Small amounts of oil, wax, antioxidant, ZnO, stearic acid, sulfur, and accelerators were included as other ingredients.

### EELS measurements

EELS measurements of pure silica and SCA were conducted to obtain reference Si-L_2,3_ spectra. A silica sample for TEM measurement was synthesized by dispersing segments of commercial silica (ULTRASIL VN 3, Evonik Industries AG) on a microgrid. The SCA specimen was synthesized from commercial SCA (Si266, Evonik Industries AG), which was diluted 100 times with ethanol. A microgrid mesh was infused with the solution and dried in a desiccator for 1 week to obtain an SCA membrane.

EELS measurements were performed using a transmission electron microscope (JEM-ARM200F ACCELARM, JEOL) operated at 200 kV. The electron source of the instrument is a cold field-emission gun. The microscope was equipped with an imaging filter (GIF Quantum ER, Gatan) for EELS measurements. The energy width of the zero-loss peak was measured at 0.5 eV. The EELS measurements were conducted at room temperature. The reference Si-L_2,3_ spectra of silica and SCA were acquired from the specimen areas 300 and 1300 nm in diameter, respectively. In these measurements, the energy dispersion of the spectrometer was set as 0.1 eV/ch, and the exposure time was 10 s. For the STEM-EELS measurements of the silica-filled SBR, the electron probe current was 480 pA, the probe was 0.2 nm in diameter, and the total acquisition time was 0.04 s per pixel. The Si-L_2,3_ spectrum overlaps with the dominant background signals due to plasmons and multiple scattering in the lower energy region. Therefore, the background intensity was subtracted by a power-law function, *AE*^*-r*^, where parameters *A* and *r* were decided by fitting the pre-edge region from 86 to 99 eV for individual spectra. EELS data analysis and MLLS fitting were performed with Digital Micrograph (Gatan Inc.)^37^.
